# Guidance Framework for Developing IoT-Enabled Systems’ Cybersecurity

**DOI:** 10.3390/s23084174

**Published:** 2023-04-21

**Authors:** Hezam Akram Abdulghani, Anastasija Collen, Niels Alexander Nijdam

**Affiliations:** Centre Universitaire d’Informatique, Geneva School of Economics and Management, University of Geneva, Route de Drize 7, CH-1227 Carouge, Switzerland

**Keywords:** internet of things (IoT), security goals, security guidelines, IoT assets, IoT security level certificates, countermeasures, IoT attacks, secure IoT frameworks

## Abstract

Internet of Things (IoT) faces security concerns different from existing challenges in conventional information systems connected through the Internet because of their limited resources and heterogeneous network setups. This work proposes a novel framework for securing IoT objects, the key objective of which is to assign different Security Level Certificates (SLC) for IoT objects according to their hardware capabilities and protection measures implemented. Objects with SLCs, therefore, will be able to communicate with each other or with the Internet in a secure manner. The proposed framework is composed of five phases, namely: classification, mitigation guidelines, SLC assignment, communication plan, and legacy integration. The groundwork relies on the identification of a set of security attributes, termed security goals. By performing an analysis on common IoT attacks, we identify which of these security goals are violated for specific types of IoT. The feasibility and application of the proposed framework is illustrated at each phase using the smart home as a case study. We also provide qualitative arguments to demonstrate how the deployment of our framework solves IoT specific security challenges.

## 1. Introduction

As a result of the development of two emerging technologies, Radio Frequency Identification (RFID) and Wireless Sensor Networks (WSNs), the notion of Internet of Things (IoT) was proposed in 1999 by Kouicem et al. [1]. The fundamental goal of IoT is to smoothly integrate real-world devices into the digital realm by utilising already installed infrastructure such as switches, routers, and gateways. To this end, a number of IoT objects equipped with sensors, actuators, and connectivity protocols have been deployed in multiple domains to offer an enormous business value for customers, organisations, and governments. For instance, smart watches, smart home appliances, and smartphones are examples of IoT diverse applications, all of which were created with the goal of improving the customers’ quality of life and productivity [2]. However, the aforementioned applications and IoT in general have encountered many security and privacy problems, the common examples of which are side-channel attacks, unauthorised conversation, routing attacks, and unexpected use of IoT data [3]. Due to two key characteristics, securing IoT is a complex task in comparison with traditional cybersecurity. The first difference is the IoT objects’ variation in their size and processing power. The second difference is their connectivity capabilities. It is, therefore, possible to apply traditional security mechanisms (e.g., Advanced Encryption Standard (AES)) directly to powerful objects such as smart phones. In contrast, power constrained objects, for instance smart light bulbs, may not be able to apply such techniques directly without some modifications due to their limited resources in terms of battery life, memory storage, and computational power. To this end, a number of of solutions have been proposed in the literature and can be broadly divided into four categories: (i) gateway-based solutions [4,5,6], (ii) IoT stack-based solutions [7,8,9,10], (iii) middleware-based solutions [11,12,13], and (iv) risk-based certifications [14,15,16].

Despite the benefits of using such solutions for addressing some IoT security concerns (e.g., secure communication), they have drawbacks. For instance, using a gateway for securing IoT objects is a matter of compromise. On the one hand, it can be used to address some of security issues such as updating objects’ firmware and providing a secure key management method between IoT objects and the gateway [17]. On the other hand, it introduces a single point of failure in both security and operation. Moreover, flexibility and scalability will be reduced and hindered, as the development of a new IoT application or IoT object requires changes to be implemented into the gateway [18]. Risk-based certification solutions are trying to overcome many limitations such as the possibility of more flexible decisions to decrease time-to-market and provide all involved stakeholders with the same tools for security assessment. The approach is different in its essence as it focuses on the risk analysis and device exposure to potential threats, as well as potential consequences severity.

The absence of frameworks that generally outline accepted security and privacy policies for IoT assets (physical objects, protocols, data at rest, and software suggested in our prior work [19]), as well as their protection measures, is another contributing issue. Such guidelines and their suitable implementation techniques would pave the road for IoT stakeholders such as developers and manufacturers to build secure IoT systems by integrating such guidelines into their systems from the start. In spite of the importance of such frameworks of security and privacy guidelines for IoT to enhance its security and privacy by design, a few research studies have been proposed in this regard [20].

Nevertheless, framework-based solutions require more efforts not only to address their limitations (e.g., poor implementation see Section 2), but also to go beyond such limitations and contribute to make them more secure and reliable. To this end, we develop a novel framework by which different Security Level Certificates (SLCs) are assigned to IoT objects based on their hardware capabilities and existing protection measures. Our framework allows IoT objects to protect themselves either independently when it fulfils the top-tier SLCs or through a dependency association between IoT objects, where a lower tier SLC IoT object is indirectly protected by a higher tier SLC. A detailed explanation of our framework is presented in Section 3.

The main contributions of this work are the following:

- Mapping of IoT attacks and security goals violation.
- Identification of most common limitation of existing frameworks for IoT security and privacy.
- Formalisation of a secure IoT framework, capable to assign different SLCs to IoT objects based on their hardware and communication capabilities.
- Showcasing feasibility of the proposed framework in the context of a smart home.

The remainder of this paper is structured as follows. In Section 2, a review of ongoing security challenges, most relevant IoT attacks and the existing cybersecurity frameworks against security and privacy threats together with their limitations are analysed. Section 3 outlines our research methodology to reason the ground work for the proposed framework for securing IoT objects and presents the formal specification of the SLC framework. Section 4 features a case study to verify SLC methodology feasibility. Section 5, discusses mitigations for IoT specific attacks and threats and the limitations with the application of SLC. The concluding remarks as well as future work is described in Section 6.

## 2. Related Work

This section aims to encapsulate versatile efforts in the IoT ecosystem towards securing IoT objects. First, an analysis of the most important security challenges is provided with the initial categorisation. Second, a review on the most occurring IoT-specific attack vectors is presented with the analysis for each attack group on how the security requirements goals are violated. Finally, a survey of latest advancements in the securing IoT is proposed.

### 2.1. IoT Security Challenges

This section presents IoT-specific security challenges grouped by the authors into eight categories.

Lack of a secure development (SC1): functional requirements are the primary emphasis of both conventional software engineering procedures and IoT systems engineering techniques. With IoT systems, however, security is rarely a top priority throughout the software development process because the installation of functional features receives more attention, leaving security requirements to be addressed once the product is finished, according to El-Attar and Abdul-Ghani [21]. Hence, this type of approach is inadequate, and IoT systems must include security standards or recommendations into them from the ground up. In order to do this, the authors have already put up a thorough list of security and privacy standards for IoT assets, particularly for physical items and protocols [19].

Tight resource constraints (SC2): different hardware limitations in terms of processing speed, storage capacity, and battery life may apply to IoT gadgets. Which is why, given the hardware capabilities of some IoT objects such as mobile phones and tablets, conventional security techniques such as AES can be applied directly to those devices. For instance, according to Taleby et al. [22], the Windows 10 Mobile employs the same security features (such as the Windows Hello mechanism) as the Windows 10 and Windows 11 Operating System (OS) for personal computers to provide protection against emerging security threats. Nevertheless, ordinary IoT gadgets (e.g., presence sensors and smoke detectors) can not implement such techniques.

Features specific design (SC3): the majority of IoT items were created with specific purposes and environments in mind. Building similar defensive mechanisms for various IoT gadgets that operate in heterogeneous contexts and provide a variety of activities and services is therefore not practical. Jeongnyeo [23] established mitigation approaches for IoT devices based on three key elements: (i) functionality, (ii) attributes, and (iii) capabilities.

Changes in security requirements (SC4): depending on the state of a larger system in which an IoT device is a part, the security needs for that object may change. One might imagine that a modern car has multiple embedded smart components. The state of the car has a significant role in determining which of these components needs to be secured the most. For instance, the anti-lock braking device is the most important one when the automobile is moving. On the other hand, if the car is stationary the most crucial one is a glass break detector device [24].

Update mechanisms (SC5): the update procedures of IoT objects have a significant impact on their security. For example, an IoT object meant to receive updates locally may need less security measures than an IoT object designed to receive updates remotely. It implies that any device that needs to securely update its firmware via a network should first establish a secure channel with the server and then verify the accuracy of a new firmware image. However, when it comes to local firmware updates, just the legitimacy of the individual installing newly released firmware into the object must always be verified [25]. According to El Jaouhari and Bouvet [26], the challenges are still ranging from interoperability issues with a lack of standardisation efforts, to the actual device management and establishment of the trust chain for the secure Firmware Over-The-Air process.

Objects’ mobility (SC6): the mobility of IoT objects is one of their key characteristics, with security greatly depending on its location, whether static or dynamic. For various reasons, a dynamic object requires additional security measures in comparison to a static one. The dynamic object might be linked to unidentified assets that show up in various situations. Therefore, according to Sen [27], such object should be equipped with distinctive safeguards such as an end-to-end security to protect its communications with other objects, tamper-proofing techniques to avert physical attacks, side-channel analysis to avoid data leakage, and a secure firmware update method. Whereas the static object might constantly be connected to trusted assets, which are in charge of guaranteeing its security.

Importance of IoT objects (SC7): the importance of an IoT object affects its security. For instance, in a WSN, a sink node requires more defensive strategies than sensor nodes because it manages the entire network in addition to gathering, aggregating, and processing data from sensor nodes. The malicious WSN nodes that continuously send undesirable signals toward the sink node or a base station could, according to Yang et al. [28], halt the entire network.

Uncontrolled environment (SC8): because some IoT objects may be deployed in remote locations and left unattended, they are vulnerable to physical attacks, such as malicious manipulation of Integrated Circuits (ICs) [29]. An attacker could clone the IoT device, steal it for further research to determine their security characteristics or steal secret keys stored on it [30].

### 2.2. IoT Attacks Vector

In the state of the art, conventional security goals have been divided into three main groups: (i) Confidentiality, (ii) Integrity, and (iii) Availability, referred to as the Confidentiality, Integrity, and Availability (CIA). Confidentiality is achieved through a set of rules that limits access to only authorised objects or users. Integrity, in the context of IoT, is also of paramount importance, as it assures the accuracy and completeness of IoT data. IoT availability is an indispensable requirement as well, since it ensures the availability of IoT objects along with their data to its users. In spite of the popularity of CIA, it fails to deal with novel threats appearing in a collaborative environment [31]. Toward this end, Cherdantseva and Hilton [31] suggest a thorough set of security goals, known as the Information, Assurance, and Security (IAS) octave, by investigating a huge amount of information in the literature in terms of security. An overview of the security goals proposed by the IAS octave, along with their definitions and abbreviations in link with IoT environment is presented in Table 1.

We enumerate common attacks against IoT and investigate their violated security goals. The selection of the attacks is based on our previous work [32], cross-linked with the latest surveys [20]. More specifically, we annotate with ‘
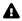
’ symbol when a security goal in question is violated by the described attack. The summary is outlined in Table 2.

Eavesdropping (AT1): intentionally listening to packets over communication links is called eavesdropping, and it is a powerful attack against communication channels if packets are not encrypted during transmission. The main goal of such attack is to intercept, read, and alter the communication packets. Three security goals, namely CONF, NREP, and PRIV, are affected by this type of attacks. The CONF and PRIV security goals are violated, since the attacker is indirectly revealing some private information by listening to communication channels that are not encrypted nor well protected. The NREP is compromised, as the attacker could recognise a private key of an object or a sender in case of a weak cryptographic algorithm and thus use such key to sign some packets and send them to other objects or recipients without revealing his/her true identity.

Physical attacks (AT2): IoT objects may be deployed in various environments where supervision of the objects is not always possible, making them susceptible to physical attacks. These attacks include, but are not limited to, vandalising circuits, modifying OS, and extracting valuable cryptographic information. In this type of attack, all security goals can be violated, as the attacker potentially has full control over the IoT object. As demonstrated by Deogirikar and Vidhate [3], not only can the attacker physically harm the IoT device, but also cause damage to a bigger IT system.

Side-channel attacks (AT3): as IoT objects execute their normal functions, there is a risk that critical information may be revealed (e.g., the secret keys). This type of attacks may happen because of the lack of secure techniques of processing and storing IoT data (e.g., storing unencrypted data directly on IoT objects). It is also worth mentioning that IoT objects may be vulnerable when not equipped with secure wireless protocols to transmit data. For example, an electromagnetic wave emitted by an object may reveal sensitive data about both the object and its users, according to [33]. Three security goals (CONF, INTG, and PRIV) are directly affected by this attack. The CONF and PRIV are violated as the attacker could reveal sensitive data about the object and its users by analysing its side-exposed features, such as algorithms and power consumption. Having discovered some security parameters (e.g., encryption keys), the attacker could modify, for instance, the transmitted data.

Malicious object insertion (AT4): maliciously adding an object to the existing set of objects by duplicating another object’s identification number to either corrupt the packets or misdirect them is the main goal of this attack. Therefore, this type of attack may cause a huge drop in the network performance, directly affecting AVAL and TRST security goals. Moreover, upon arrival of messages at a replica, an attacker could not only gain access to different security parameters (e.g., encryption keys), but also revoke authorised objects, since the attacker could execute an object-revocation protocol exposing CONF, NREP, and PRIV. In summary, this attack violates all security goals, as the attacker has capability to misdirect, drop, decrypt, and corrupt the messages.

Routing attacks (AT5): in [19], the authors illustrate several attacks such as Gray hole, sybil, and worm hole designed specifically to target how IoT packets are directed. The consequences of such attacks include, but are not limited to, dropping, spoofing, and misdirecting packets. The simplest form of such attacks is known as modifying attack in which routing information is illegally manipulated by an attacker. The CONF, INTG, and PRIV security goals are violated as the attacker is indirectly capable of disturbing routing paths and spoofing packets. ACNT is also affected as the attacker could drop or misdirect some messages. Finally, NREP and ACNT are endangered as the attacker has a capability to disrupt the delivery of the packets.

Malicious firmware (AT6): several manufacturers such as Apple and Sony have been using Over-the-air (OTA) methods to update their objects which were already being deployed in power grids, smart homes, smart cars, and more. Due to the large number of IoT objects that require updates, a trusted server has been used by manufacturers to publish or push newly released updates of their objects. This method, however, is vulnerable to a single point of failure because of Denial of Service (DoS) attacks and a huge number of valid update requests sent simultaneously to the server. This attack violates all security goals as the attacker has full control over IoT objects.

### 2.3. Mitigation Techniques

The following section presents the summary and classification of existing mitigation techniques relevant for the selected attack vector presented previously. Table 3 outlines the analytical correlation between each mitigation technique and related attack.

Link layer security (MT1): IP-based communication in IoT is mainly reliant on IPv6 networking for Low power Wireless Personal Area Networks (6LoWPAN) [34], which is dependant on the IEEE 802.15.4 link layer and provides hop-to-hop security. It implies, that each object in the communication link should be trusted without authentication, as well as key management, time-synchronised communications, and reply protection. To address the lack of reply protection as well as time-synchronised communication, the IEEE 802.15.4e extension (modification) was introduced in 2012 by the IETF [35]. It is critical to understand that link layer security cannot safeguard packets once they leave its network. Several security solutions have been offered to address this issue. Roman et al. [36] suggest a wireless sensor network key management system. This type of solution increases security at the link layer. According to ArchRock Corporation [37], PhyNET secures a link between a border router and nodes using IPsec in a tunnel paradigm. Transport layer security (MT2): end-to-end security can be provided by both Transport Layer Security (TLS) and Secure Sockets Layer (SSL). Because they enable authentication, key exchange mechanisms, confidentiality, and integrity, these systems have been widely utilised to secure communications over the traditional Internet. TLS and SSL, however, cannot be utilised directly for IoT for two reasons. First, TLS is used over TCP, which is not an appropriate approach for IoT gadgets due to their restricted resources. Second, TLS/SSL session establishment and key exchange necessitate a series of packet exchanges. SSL and TLS, on the other hand, have been recommended as IoT security solutions. Hong et al. [38] presented an SSL-based security solution for smart objects. According to their findings, a full SSL handshake, including packet exchanges, takes 2 s to complete. Datagram Transport Layer security (DTLS) is introduced to provide security means similar to TLS; however, it is built on top of UDP. Kothmayr et al. [10] present a two-way authentication mechanism for IoT, which is strongly reliant on existing Internet standards, particularly the DTLS protocol. This technique was implemented through the exchange of x.509 certificates containing RSA keys and an authenticated DTLS handshake.

Network layer security(MT3): these methods are divided into two categories: 6LoWPAN and Routing Protocol for Low-Power and Lossy Networks (RPL). The IETF has standardised 6LoWPAN as a network layer protocol. It allows Internet access for resource-constrained objects thanks to a header compression method. 6LoWPAN, on the other hand, does not offer security mechanisms or key management. Kothmayr et al. [10] present unique compressed security headers appropriate for 6LoWPAN to provide end-to-end network layer security. Such security headers make it easier to integrate 6LoWPAN with IP Security architecture. Raza et al. [39] propose an IPsec extension appropriate for 6LoWPAN to provide IPsec-based security for IoT items. In terms of energy usage, processing time, and packet size, 6LoWPAN/IPsec is a suitable solution for securing IoT items, as opposed to link layer security. RPL is a network layer protocol that is also IETF-standardised. It explains the RPL packets sent over ICMPv6 between Low-Power and Lossy Network (LLN) objects. Within the LLN, these packets constitute a routing table. The RPL specification defines three types of security: unsecured, authenticated, and preinstalled.

Firmware update methods (MT4): are either remote or direct. A server node broadcasts the availability of a new version of a firmware for remote update. The announcement of the update is forwarded, by any node with the latest update, to all nodes in its vicinity. Nodes compare their current firmware to the new version and initiates the upgrade, if needed, with the advertiser. For security, all requests, answers, and data packets should be authenticated and encrypted. Law et al. [40] point out specifically that possible disruptions from DoS attacks should be dealt with at each stage of this complex process. Lastly, an end user attempting to install manually a firmware should be authorised and authenticated.

Intrusion detection system (MT5): the primary goal is to ensure that general policies are not violated through the usage of a continual monitoring procedure. By tracking aberrant requests to objects, it gives a reliable approach to counteract both battery-draining and sleep deprivation attacks. Saiful Islam Mamun et al. [41] reflects on the continuing research for monitoring edge nodes and counteract potential attacks at this level.

Side channel protection (MT6): provides an effective approach for detecting both hardware Trojans and malicious software on IoT devices [42]. The presence of a Trojan in an IoT object or circuit affects its components, the most frequent of which being power and gates and has the potential to alter heat distribution on the IC. The survey from Sadhu et al. [20], highlights the feasibility of detecting rogue firmware through side-channel analysis.

Decommissioning methods (MT7): eventually IoT objects will reach a point when they must be decommissioned; thus, these objects must be withdrawn and cannot be reintroduced to the network. Notwithstanding the relevance of decommissioning in addressing various security and privacy issues, there has been little research and development in this area. Smart Card Alliance [43] has proposed two options for decommissioning. To begin, the objects can be reset to their factory default settings. Apart for the minimum security parameters, this option deletes all data in such objects. The second option is to prevent blocked objects from re-joining a network until their statuses on the server have been updated.

Secure bootstrapping (MT8): Heer et al. [44] state the importance of the architecture impacting the secure bootstrapping technique implementations. Using a Diffie–Hellman algorithm, two IoTs can agree on a shared secret in a distributed architecture. Numerous protocols, including TLS, DTLS, Host Identity Protocol (HIP), and IKEv2, can be used to complete a key exchange and set up security parameters without a trusted party. Nonetheless, putting such methods into practice on severely limited objects is quite challenging. Many research initiatives have been suggested as solutions to this problem, including Diet HIP [45] and human memorable passwords, which build trust relationships between IoT products and gateways [46].

Blockchain solutions (MT9): aim to build transactions or communications between objects in a distributed architecture without the requirement for centralised trust entities, and they has influenced the world of cryptocurrencies. Once a transaction is validated using such technology, it cannot be disputed. Notwithstanding the advantages of the blockchain, its integration into the IoT has a number of obstacles that must be overcome, such as bandwidth consumption, partial anonymity, tremendous processing capabilities examined by [47], and most crucially, time latency.

Hardware-based solutions (MT10): according to Mosenia and Jha [48] altering the circuit is one of the best defences against physical, side channel, and Trojan attacks. Employed countermeasures against side-channel assaults are shielding, adding randomised delay and noise. Tamper-proofing mechanisms may be added to IoT products to increase protection against physical attacks. Lastly, Hristozov et al. [49] describes a promising hardware-based run-time attestation approach, whereby an item attests its firmware by a remote entity.

Deduplication schemes (MT11): enforce redundant IoT data is be kept once, and links to the duplicates—not the copies themselves—are provided. Because of this, such an approach can be employed as a fallback plan [50]. Hence, it is both necessary and difficult to build safe deduplication techniques that can identify identical data copies and store them just once. In order to do this, a number of data deduplication strategies have been put forth in the literature. Based on the location at which data deduplication is completed, these techniques can be broadly divided into two categories (server-side and client-side) [51].

Anonymisation schemes (MT12): k-anonymity, l-diversity, and t-closeness are the three major categories. K-anonymity is a strategy that protects data holders’ privacy when they release their data. It ensures that each person’s information cannot be recognised from a group of at least k(-1) persons. L-diversity is proposed to reduce K-anonymity inability to avoid both homogeneity and background attacks. Machanavajjhala et al. [52] presented a l-diversity privacy strategy that may be used to prevent a variety of assaults (e.g., homogeneity attack). Furthermore, they conduct an experimental assessment to demonstrate that the suggested approach is realistic and can be effectively applied. Li et al. [53] proposed the term t-closeness to address the inadequacies of k-anonymity and l-diversity related with attribute inspiration. The authors recommended that the distribution of sensitive information in each set must be close to or connected to the dispersion of sensitive information in the whole database.

Transient data storage (MT13): few studies have focused on handling transitory IoT data created during system executions. The significance of transitory data originates from the processing of data during system execution to form new data views, which may be maintained in storage for user requirements or discarded, and therefore it may lessen hazards connected with such data. Narendra et al. [54] suggested a method for handling transitory IoT data that allows such data to be processed, stored, and maintained.

Secure storage schemes (MT14): may be used to prevent IoT data breaches and are divided into two types: cryptographic and non-cryptographic techniques. Jiang et al. [55] provides an example of a cryptographic-based system based on Shamir’s secret sharing mechanism for storing data. Storer et al. [56] presented a non-cryptographic approach, introducing POST-SHAREDS, a storage format that provides long-term security for IoT data without the need of encryption methods. The security of such a strategy stems from separating data into so many segments and dispersing it over several storage locations.

Searchable encryption (MT15): from the domain homomorphic encryption, another method for protecting data in IoT storage is to conduct information retrieval on encrypted data, known as Searchable Encryption (SE). The basic principle is that an object should index and encrypt its data before sending it, along with an index, to a server. To search for data, the object must produce a trapdoor via which the server may directly run search operations on encrypted data, and encrypt its output as well.

Monitoring and auditing (MT16): is crucial, especially when it comes to preventing data breaches. In order to monitor servers, agents, files, and their configurations, Anand [57] have presented a centralised monitoring strategy for cloud applications. This technique offers multi-level notifications, redundancy, and automated recovery to overcome the drawbacks of a centralised monitoring approach, which include scalability and, most critically, a single point of failure. A scalable monitoring system for clouds has been put forth by Brinkmann et al. [58], proposing a sparse management tree that includes a number of parameters and their data gathering protocols. The authors also examine the drawbacks of current intrusion detection technologies and look at the potential of virtual machine level intrusion detection.

Recovery strategy (MT17): despite the significance of providing high availability and disaster recovery for IoT storage, the state of the art only has a few research suggestions. The issue of uploading IoT data from a collection of various sensors and the production of various replicas of this data on distributed storage in the cloud has been examined by Kumar et al. [59]. The availability of numerous distributed data centres, sometimes known as mini-clouds, is a prerequisite for data recovery strategies.

Access control methods (MT18): can be categorised into four groups: (i) Attribute-Based Encryption (ABE), (ii) Discretionary Access Control (DAC), (iii) Mandatory Access Control (MAC), and (iv) Role-Based Access Control (RBAC). The system administrator will have the ability to control the responsibilities and rights of the customers after integrating MAC into an IoT system. Further allowing the system administrator to alter access policies and denying users access to the network. Sensitive systems, such as those used by the military and research institutions, can include this kind of access technique [60]. Customers will be able to change the access rules for any items if DAC is integrated into an IoT system. If an attacker is able to access a client account, this strategy is quite risky. As a result, giving a consumer complete access to the IoT system is not a good idea. Customers can acquire access to resources based on their roles and responsibilities in the system if RBAC is implemented into an IoT system. ABE enables flexible one-to-many encryption without knowing who would access the information. It also highlights the fine-grained access approach for outsourced data. In ABE, a customer is identified by a collection of attributes that may be used to determine the client’s access policy.

Secure IoT OSs (MT19): designing and building a specific IoT environment OS is critical for providing object security at all levels. Javed et al. [61] conducted an in-depth analysis of existing techniques and validated security as a missing component that must be addressed immediately. Their assessment verified open problems, such as the provision of data integrity, authentication, and access procedures.

SDN-based solutions (MT20): primary goal of such technology is to separate the network control plan from the data plan. This type of separation would allow for dynamic network administration, centralised setup, and network control [1]. Objects (e.g., routers, gateways, and switches) in the SDN paradigm cannot make control choices (e.g., forwarding tables), but they may learn such decisions from a centralised entity known as an SDN controller. SDN is a viable approach for addressing various IoT security concerns due to its centralised design.

Application layer security (MT21): depends heavily on the needs of the individual IoT system and application protocol. MQ Telemetry Transport (MQTT) and Constrained Application Protocol (CoAP) are the most relevant in terms of data collection in this context, whereas Advanced Message Queuing Protocol (AMQP), Data Distribution Service (DDS) and Extensible Messaging and Presence Protocol (XMPP) are appropriate for specific service requirements, namely business messaging, instant messaging, online presence detection, and real-time exchanges [61]. Aside from issues related to improving application layer security with CoAP, several research works have addressed some common issues such as the lack of mapping techniques between TLS and DTLS, the absence of digital certificates and public-keys, and, most importantly, the enforcement of object security with CoAP [62].

### 2.4. Framework-Based Solutions

In this section, we review the existing frameworks of security and privacy guidelines along with their shortcomings. Although the development of a comprehensive set of security and privacy guidelines, covering all IoT assets, is currently an indispensable requirement for building secure IoT systems, a few frameworks equipped with such guidelines have been proposed, which we briefly present in the following paragraphs.

Perera et al. [63] suggest a list of privacy guidelines for IoT middleware and applications and their data at rest. Such guidelines include, but are not limited to, reducing data granularity, blocking repeated queries, and distributing data storage. However, they do not propose guidelines for different IoT assets such as physical objects (computing nodes and RFID), protocols, and OSs. Moreover, they do not address attacks and threats against IoT, nor do they identify suitable protection measures to implement their guidelines.

In [64], the Broadband Internet Technical Advisory Group (BITAG), suggests an abstract list of security and privacy guidelines (e.g., encrypting communications) for some of IoT assets (computing nodes, applications, and protocols). That said, BITAG neither provides a thorough set of guidelines, nor do they recognise proper security mechanisms to carry out the guidelines. Moreover, attacks and threats against IoT are left untouched.

Open Web Application Security Project (OWASP) proposes a list of security and privacy guidelines for some IoT assets (computing nodes, applications) [65]. Nevertheless, the OWASP does not identify attacks and threats against IoT, nor does it discuss the required security techniques to apply its guidelines.

Abdulghani et al. [32] propose a comprehensive list of security and privacy guidelines only for IoT data at rest, such as searching on encrypted data, ensuring authorised access, encrypting data storage, and minimising duplicated copies. Moreover, the authors investigate all possible attacks and threats against data at rest and identify a set of protection measures which can be used to implement their guidelines. Moreover, they show the link between their guidelines, attacks, and mitigation techniques.

In [66], the IoT Security Foundation (IoTSF) proposes a complete list of security and privacy guidelines for all IoT assets, except RFID tags. Nevertheless, IoTSF does not address attacks and threats against IoT, nor does it distinguish suitable implementation techniques to accomplish its guidelines.

A comprehensive list of security and privacy guidelines for some IoT assets (computing nodes, RFID, and protocols) is proposed in [19]. The authors also investigate all possible attacks and threats against them. Furthermore, they identify proper protection measures to implement their guidelines. Not only that, they also show the link between their proposed guidelines, attacks, and protection measures.

In [23], the authors first state the importance of defining security requirements for IoT objects based on three factors: (i) functionality, (ii) capabilities, and (iii) characteristics. Then, they investigate security threats as well as vulnerabilities of IoT objects, and more importantly they utilise the classification of IoT objects capabilities into different classes to suggest a list of security requirements suitable for each class.

Risk-based security certification is conceptually distinct from existing methods used to address security and privacy issues in the IoT ecosystem because it changes the emphasis from verifying the precise security level to the possible exposure to security vulnerabilities. Baldini et al. [14] have provided a certification framework aiming to address the shortcomings of existing Common Criteria certification scheme based on ISO/IEC 15408 standard. The proposed certification process is composed of several steps, ranging from risk analysis and labelling, vulnerability patterns identification to the execution of the test suites. However, in comparison with previously presented frameworks, this approach is prone to be domain specific and heavily depends on the operational context to generate the necessary models and tests.

In a similar direction of the risk-based IoT labelling, Matheu-García et al. [15] have proposed a security certification methodology targeting all stakeholders to be able to access the security solutions based on ISO 31000 and ISO 29119. The developed framework demonstrated its applicability in a scenario on automation of security testing with corresponding benchmarking analysis. The focus of such methodology, similarly, is also given to the vulnerability analysis and correlation with a profile or security label. The scope of the common attacks shielding is not explicitly referenced, nor are the targeted guidelines for security and privacy issues provided.

Table 4 summarises the recently-published frameworks that suggest several security and privacy guidelines for several IoT assets along with their appropriate implementation techniques.

It can be observed that the suggested research proposals presented in Table 4 suffered from one common limitation which is the lack of a list of security and privacy guidelines that cover all IoT assets. Moreover, the authors in [32] have stated that the success of such frameworks of security and privacy guidelines depends heavily on the their implementation techniques. Poor implementation of such frameworks, therefore, may lead to develop insecure IoT systems despite of having security and privacy guidelines.

We do believe that framework-based solution is the answer to many security challenges now facing IoT to reach its full potential. This is because such frameworks have suggested a set of security and privacy guidelines along with their protection measures which can be utilised by different IoT stakeholders (e.g, developers and manufacturers) to build secure systems from the ground up. This kind of practice will definitely enhance security and privacy by design for IoT.

## 3. Methodology

**Figure 1:**
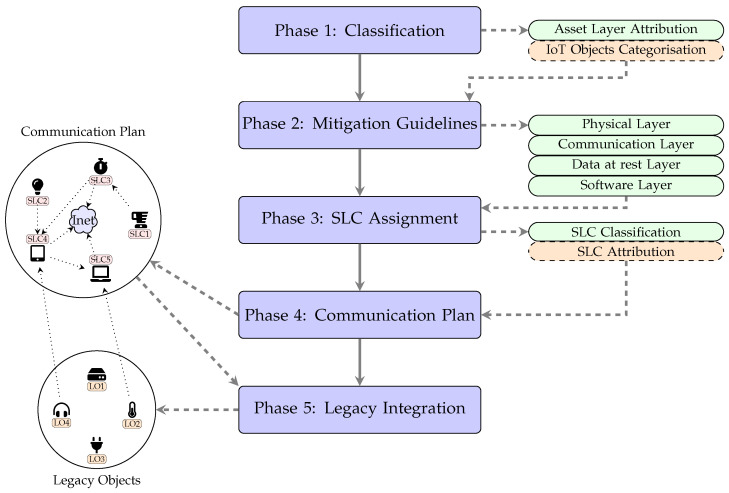
An overview of the proposed methodology.

This section is dedicate to the proposed methodology through which different SLCs are assigned to IoT objects based on their hardware capabilities as well as their implemented protection measures such as Intrusion Detection System (IDS), side channel protection, and secure storage schemes. IoT objects equipped with SLC1 or SLC2 indicate that they will have weak protection measures (e.g., Data Link Layer Security (DLLS)) and limited hardware resources. Hence, these objects will neither be deployed in unattended areas, nor will they be connected directly to the Internet. Such objects will depend heavily on objects with SLC3 (acting as gateways) to protect them and manage their communication to the Internet. In contrary, IoT objects armed with SLC3 or SLC4 or SLC5 indicate that they will have strong protection mechanisms (e.g., blockchain-based solutions) and powerful hardware resources. These objects, thus, can be deployed in uncontrolled environments and more importantly can be connected directly to the Internet and protect themselves autonomously. Moreover, Security Level Certificates Framework (SLC Framework) states the communication plan in which different IoT objects can communicate securely with each other or with the Internet based on their SLCs. The proposed framework consists of five main phases, which are described below. An overview of the overall methodology is depicted in Figure 1.

### 3.1. Phase 1: Classification

In this phase, IoT assets along with their associated attacks and countermeasures are attributed a specific layer, enabling association to relevant attack vector. At the second step, each IoT object is classified into different category based on their hardware capabilities.

#### 3.1.1. Asset Layer Attribution

Due to the complexity of IoT ecosystem composed of so many enabler technologies (e.g, WSN and 6LoWPAN), it is essential to recognise precisely IoT assets to be able to protect them. To contribute to such objective, many IoT Reference Models (RMs) have been proposed in the literature, such as a three-level model [67], a five-level model [68], and a seven-level model [69]. Even though such RMs simplify the complexity of IoT by breaking it into different layers, they do not address the required building blocks for their layers or levels, which can be used by IoT developers to easily construct their systems. Toward this end, a novel building-blocked RM for IoT was introduced in our earlier work in [19], and IoT assets were divided into four main component layers:

- Physical layer: consists of computing nodes (RFID readers and sensor nodes) and RFID tags.
- Communication layer: includes all IoT protocols covering all IoT stack and the existing network infrastructures (e.g., routers and switches).
- Data at rest layer: involves data stored either in IoT objects or on the Cloud Storage Service (CSS)
- Software layer: is composed of IoT middleware, IoT applications, and IoT OSs.

It is worth mentioning that the process of identifying all possible attacks and threats against each IoT asset and also recognising their suitable protection mechanisms has been already investigated in the same work. Asset layer attribution procedure is static and is performed once for the initial classification.

#### 3.1.2. IoT Objects Categorisation

In the IoT environment, different types of IoT objects that vary from tiny and lightweight objects (e.g, light bulbs) to powerful objects (e.g, smart phones) are being connected to the Internet or each other to achieve specific tasks. It is therefore unwise to suggest common implementation techniques for all of them, since some objects, for instance light bulbs, would not be able to run them due to their limited resources. Therefore, there is a need to classify IoT objects into different categories based on their hardware capabilities. SLC Framework classifies IoT objects into five categories based on four primary factors: (i) Central Processing Unit (CPU), (ii) memory, (iii) power consumption, and (iv) on-board data storage.

Each factor has a specific weight criteria, and is given a priority in the presented order of appearance. SLC Framework dictates that Category 1 IoT object has minimal hardware capabilities, while IoT object of Category 5 has very powerful processing equipment. IoT objects categorisation procedure is use-case specific and must be tailored for each ecosystem to which framework is applied. An overview of objects classification along with a real example for each category is presented in Table 5.

### 3.2. Phase 2: Mitigation Guidelines

In this phase, a set of security and privacy guidelines are proposed for each asset layer, attributed in Phase 1.

#### 3.2.1. Physical Layer

This layer is susceptible to several attacks and threats, the most popular of which are physical attacks, object replication attacks, side-channel attacks, and Hardware Trojan attacks. The guidelines specific to this layer can be utilised at the early stages of systems development life cycles. For example, one guideline recommends that a hardware secure boot process should be integrated into each IoT object. If such a guideline is implemented by developers or manufacturers, it will prevent such object from running malicious code. Table 6 highlights the suggested security and privacy guidelines together with the reasoning and recommended mitigation implementation techniques, based on our previous research [19].

#### 3.2.2. Communication Layer

Similarly, this layer is vulnerable to several attacks including, but not limited to, side channel attacks, malicious packet modification, routing attacks, malicious node injection, and eavesdropping. In general, it is advised to equip each IoT object with mitigation techniques such as Network layer security, Application layer security, and transport layer security, to prevent malicious packets modification. An overview of the proposed guidelines for communication layer, along with their purpose and protection measures is presented in Table 7.

#### 3.2.3. Data at Rest Layer

Data at rest either on IoT objects or in the cloud is also susceptible to different attacks, such as misuse of data remnants, linkage attacks, data manipulation, insider attacks, side-channel attacks, and homogeneity attacks. To lessen such attacks, security and privacy guidelines can be implemented by either IoT developers, manufactures, and providers from ground up so that IoT data stored by their applications is fully protected. The prevention of IoT data leakage, for instance, requires IoT stakeholders to implement a set of techniques into their systems at the start such as monitoring and auditing schemes, SE, anonymisation schemes, and transient data storage. Table 8 outlines our suggested guidelines at this layer, along with their reasoning and mitigation implementation techniques, based on our previous findings [32].

#### 3.2.4. Software Layer

This layer, composed of IoT application, OSs, and middleware, is also vulnerable to many attacks and threats such as DoSs attacks, Structured Query Language (SQL) injection attacks, weak authentications, malicious requests, and viruses. Having integrated access control mechanisms into IoT applications, OSs, and middleware, such software will be able to prevent malicious requests coming from either adversaries or malicious objects. A summary of our proposed guidelines for this layer, along with their purpose and protection measures is presented in Table 9.

### 3.3. Phase 3: SLC Assignment

In this phase, an outline of SLCs classification methodology used in SLC Framework is presented, which is a static procedure common to any SLC Framework application. However, the second procedure, the assignment of SLCs to IoT objects, is use-case specific and dynamic by its nature.

#### 3.3.1. SLCs Classification

Classifying SLCs is a fundamental requirement, and it stems from two primary reasons. One is that IoT objects come in different sizes and hardware capabilities. In general, most of IoT objects have limited resources, but this is not always the case since some objects may have powerful hardware resources. Thus, it is impractical to assign common mitigation techniques for all IoT objects. The other reason depends on the environment at which IoT objects are being deployed and operated. Objects operating in a controlled area will require less protection measures as they will always be connected to trusted objects and will always be monitored by human beings or security cameras. On the other hand, objects operating in an uncontrolled environment will need more protection measures, since they will neither be connected to trusted objects, nor will be monitored by human beings or security cameras. To this end, all protection measures suggested to implement our proposed guidelines for previously mentioned IoT assets are classified into five groups known as SLCs, starting from SLC1 to SLC5. The number attached to each SLC indicates its security level, which is very weak at SLC1 and very strong in SLC5. This is because SLC1 includes only two protection measures (MT1: Link layer security and MT8: Secure bootstrapping), whereas SLC5 includes almost all mitigation techniques. It is worth noting that the process of assigning and issuing SLCs depends heavily on entities (e.g., IoT manufacturers or IoT developers) that implement our framework. More importantly, it is assumed that such entities are trusted and authenticated so that they will neither issue nor assign fake SLCs. An overview of SLCs classification used in our suggested framework along with their mitigation techniques is presented in Table 10.

SLC1: this type of SLC implements two mitigation techniques, MT1 and MT8, namely link layer security and secure bootstrapping. Thus, IoT objects with SLC1 are able to encrypt and decrypt packets only at data link layer because of DLLS. In other words, they are capable to provide hop-to-hop security and join or rejoin gateways securely (objects with SLC2) due to their secure bootstrapping techniques. Lacking hardware-based solutions to prevent physical attacks and end-to-end security techniques (e.g., Transport layer security) to provide secure communication channels, objects with SLC1 can neither be deployed in an uncontrolled environment, nor can be connected to the Internet directly. Furthermore, objects with SLC1 are not able to store data locally as they lack secure methods of storing IoT data. They also are not able to update their firmware autonomously since they do not have secure firmware update methods, and they depend on objects with SLC2 to do so. Moreover, objects with SLC1 depend on objects with SLC2 to register their SLCs in objects with SLC5, responsible for tracking of SLCs for all objects.

SLC2: has five mitigation techniques, such as MT1: Link layer security, MT2: Transport layer security, MT3: Network layer security, and MT4: Firmware update methods. IoT objects with SLC2 have capabilities to encrypt and decrypt packets at data link, transport, and network layers. That said, IoT objects with SLC2 can only communicate with the Internet through objects with SLC3, as objects with SLC2 do not have side channel protection measures to prevent data leakage and IDSs to detect malicious packets. In other words, such objects can not connect directly to the Internet. Since such objects have firmware update methods, they are be able to update their firmware by connecting objects with SLC4, which are responsible for managing firmware updates inSLC Framework. Like objects with SLC1, objects with SLC2 are not deployed in an uncontrolled area because they lack required security techniques (e.g., tamper-proofing methods) to prevent physical attacks. Furthermore, objects with SLC2can not store data locally due to the absence of secure techniques to do so. Unlike objects with SLC1, objects with SLC2 are capable to register their SLCs in objects with SLC5 and also communicate with other objects securely, as they have end-to-end security techniques (e.g., Network layer security and Transport layer security).

SLC3: this type of SLC is integrated with 14 mitigation techniques such as MT2: Transport layer security, MT4: Firmware update methods, MT5: Intrusion detection system, and MT19: Secure OS. IoT objects with SLC3 are able to connect directly to the Internet and also manage communication between objects with SLC1 and SLC2 and the Internet. This is because objects with SLC3 are armed with the required protection measures such as end-to-end security techniques, side channel protection methods, secure OSs, and more importantly IDSs to detect malicious packets. Unlike objects with SLC1 and SLC2, IoT objects with SLC3 can not only to deploy and operate in unattended areas, but also to update their firmware by connecting objects with SLC4. Furthermore, IoT objects with SLC3 store their data or data coming from objects with SLC2 temporally on their data storage after simple data processing to offer quick response to objects with SLC2, as they are equipped with transient data storage techniques. Nevertheless, IoT objects with SLC3 lack secure data destruction as well as recovery mechanisms. To this end, such objects store data just for a short period of time (e.g., per an hour). However, such objects communicate with objects with SLC5 to store their data for a long time, as they have suitable protection measures (e.g., secure storage sachems, recovery strategy, and deduplication schemes) to prevent data at rest breaches.

SLC4: this type of SLC will be equipped with 15 protection measures. IoT objects with SLC4 are responsible for managing firmware updates of the all IoT objects equipped with different SLCs. More importantly, objects with SLC4 utilise blockchain-based solutions such as smart contracts proposed in [85] to manage secure IoT firmware updates for all IoT objects participating in SLC Framework. As a consequence, manufacturers, who implemented the framework, can create smart contracts for the newly-developed firmware versions and push them to all objects with SLC4. Having pushed the smart contracts to the blockchain network formed by different objects equipped with SLC4, objects with SLC2, SLC3, and SLC5 are able autonomously query objects with SLC4 and therefore download the latest versions of firmware available for them. That said, time latency to register each smart contract to the blockchain network may take a long time (e.g., 10 min per transaction), but this is not an issue since objects’ firmware updates may be released once per month or week. As objects with SLC4 are used to store the latest versions of objects’ firmware and are equipped with secure decommissioning methods to securely destruct their data when reaching their end-of-life stages to prevent data breaches (e.g., misuse of data remnants).

SLC5: this type of SLC includes all our suggested mitigation techniques, except transient data storage and IDS. As IoT objects with SLC5 are equipped with blockchain-based solutions, such objects are responsible for registering and tracking of all SLCs into their chain. Such objects, however, are not responsible for assigning and validating SLCs, since this process will be archived by entities implemented our framework from the early stages of their systems development processes. Each object in the framework (except object with SLC1) have to register its SLC in object with SLC5. This step is an indispensable requirement for many reasons. One is that objects are not able to change their SLCs, since they are allowed to register their SLCs once in objects with SLC5. Another reason is that it eases the communication process among objects with different SLCs, as their public keys will be accessible by all the objects (except objects with SLC1). The other reason is that it detects malicious objects with fake SLCs, as each object is able to verify the SLC of another object by checking its SLC in the chain in objects with SLC5. More importantly, fake objects are placed in a revocation list by objects with SLCs5, and all objects (except object with SLC1) are then notified to stop communication. Like objects with SLC4, objects with SLC5 are able to deploy and operate in uncontrolled environments, and can destroy their data in a proper way. Unlike objects with SLC4, these objects will provide a recovery strategy of their data, and most importantly are responsible for integrating legacy objects.

It is clear that the framework provides a common method by which all objects (except objects with SLC1) are able to communicate with each other securely. This is because objects with SLC3, SLC4, and SLC5 implement the same protection measures in Transport layer security and Network layer security.

Furthermore, cost-effectiveness is an important factor when it comes to securing IoT devices from cyberthreats and implementing appropriate countermeasures, especially since these systems frequently have limited hardware resources. To make the most use of limited resources, countermeasures must be prioritised depending on their effectiveness and resource requirements. Resources can be allocated more efficiently by focusing on the most critical countermeasures. Furthermore, it is encouraged to employ lightweight security protocols that do not create an undue cost on the system. Protocols such as TLS, lightweight cryptography, and CoAP are excellent examples of such lightweight security measures. Finally, implementing security by design is crucial for ensuring that security is an integral part of the IoT system. By incorporating security from the design stage, countermeasures can be implemented without introducing significant changes to the system, which can be costly and time-consuming. By adopting these strategies, IoT systems can better balance the need for security with resource constraints.

#### 3.3.2. SLC Attribution

As we have classified IoT objects based on their hardware capabilities into five categories (see Table 5) and SLCs based on their mitigation techniques into five levels (see Table 10), it is crucial to present the link between them. SLC Framework defines that IoT objects in Category 1 have limited hardware resources, therefore they can only implement SLC1, as it has only two mitigation techniques. However, such objects will not be able to implement SLC3, or SLC4, or SLC5 due to their limited resources. Similarly, IoT objects in Category 2 are capable to implement either SLC1 and SLC2, as they have required hardware capabilities to do so. Unlike objects in Category 1 and Category 2, objects in Category 5 have powerful hardware resources to implement any one of SLCs.

It is worth stressing that the process of assigning SLCs to IoT objects must be performed with caution. This is because the improper assignment of SLCs to IoT objects may lead to instantiation of insecure objects despite having strong hardware capabilities. For instance, assigning SLC1 or SLC2 to objects in Category 5. Table 11 states the SLCs that are suitable for each category.

#### 3.3.3. SLC Implementation

In regard to the implementation and to address security concerns in IoT devices with limited memory resources, it is necessary to integrate SLCs within the firmware of devices that have low hardware capabilities for IoT objects identified as SLC1 to SLC4. However, for SLC5 devices which have strong hardware capabilities, the security level assignment can be added during the framework deployment by the developer. This allows for a more efficient use of memory resources within IoT devices and ensures that they are able to communicate securely with other devices in the network or with the Internet.

### 3.4. Phase 4: Communication Plan

Defining a communication plan between objects, in our framework, depends heavily on their SLCs to minimise the risks associated with weak links and also reduce unexpected used of IoT data. To this end, object with SLC1 can only communicate with objects with SLC2 due to their weak protection measures. Not only that, such objects are not able to communicate with objects having the same SLCs as these objects may have different link layer protocols (e.g, IEEE 802.15.4 and Bluetooth). To do so, they depend on objects with SLC2 to perform a required translation between these protocols. Unlike objects with SLC1, objects with SLC2 can communicate with all objects in the framework as long as objects with SLC3, SLC4, and SLC5 operate the same algorithms or mechanisms implemented by objects with SLC2, in their Transport layer security and Network layer security. Nevertheless, such objects can not communicate with the Internet without using object with SLC3. Unlike objects with SLC1 and SLC2, objects with SLC3, SLC4, and SLC5 can communicate with the Internet and more importantly communicate with all objects easily (except objects with SLC1). Table 12 summarises the proposed communication plan among IoT objects equipped with SLCs.

### 3.5. Phase 5: Legacy Integration

The integration of legacy objects, i.e., IoT objects not supporting the SLC Framework, with supported objects is a fundamental requirement towards achieving compatibility in IoT. Toward this end, we propose a method that will allow legacy objects to communicate with supported objects in a secure manner.

**Figure 2:**
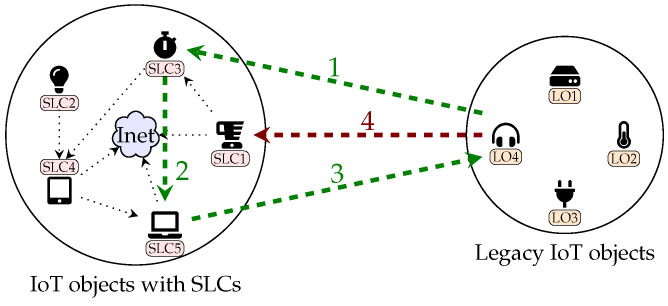
Integration the legacy objects with our framework.

Figure 2 illustrates an example on the integration of the legacy objects with objects developed according to our suggested framework. First, a legacy object, if it in the range of the network, tries to communicate with the SLC Framework’ objects, except for objects with SLC1 and SLC2 due to their restricted communication plan (illustrated with arrow 4 in red). Second, upon receiving the request from the legacy object, our object first checks if that object has a SLC. If not, it sends the request to any objects with SLC5, as they are responsible for integrating legacy objects with our objects (illustrated by arrow 1 and 2 in green). Third, an object with SLC5 suggests a set of algorithms that should be implemented in a transport layer of the legacy object to enable communication with other objects with an end-to-end security (arrow 3 in green). If the legacy object lacks such algorithms or is not able to implement them, this legacy object is rejected. In summary, a legacy object is able to communicate with objects with SLC3 and above as long as it uses the same protection measures in its transport layer implemented by those objects.

## 4. Case Study: Smart Home

This section illustrates how SLC Framework can be utilised to develop a secure smart home system as a simplified case study to present more clearly the benefits of the suggested framework. Nevertheless, the proposed methodology is domain agnostic, and thus it can be applied in other IoT domains. SLC Framework consists of static and case-specific procedures, and validation of the second type of procedures is demonstrated below through the presentation of the necessary execution steps to be performed by IoT developers or software engineers.

### 4.1. Step 1

**Figure 3:**
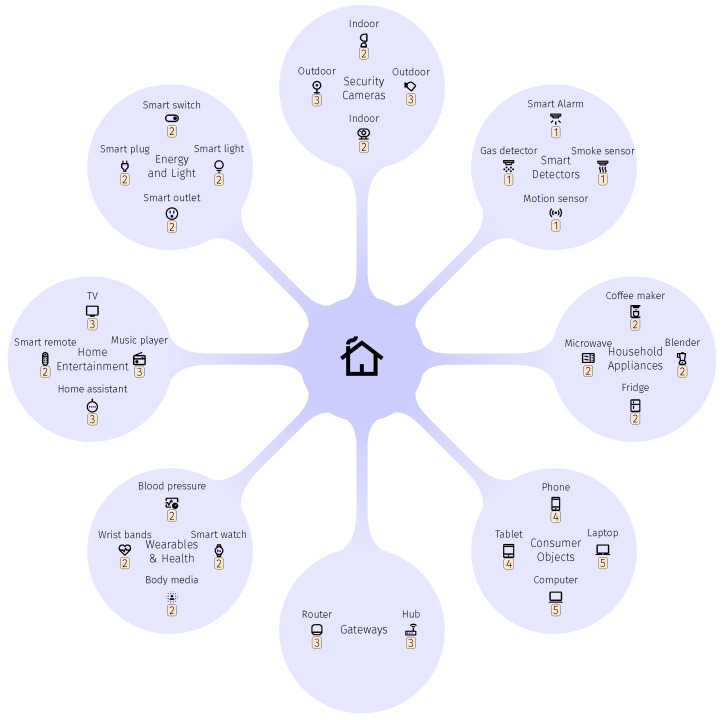
Smart home objects’ categorisation.

First, all IoT objects operating in smart homes are identified and then classified according to Phase 1 of SLC Framework. With the advent of IoT vision in which most of the physical objects around us are connected to the Internet, the number of IoT objects functioning in smart homes will be almost endless [19]. Such objects include, but are not limited to, light-bulbs, smart switches, microwaves, dishwashers, TVs, projectors, and smart phones. To this end, we classify smart home objects into eight groups such as smart detectors, household objects, and consumer objects. Furthermore, each object is attributed category level, as per IoT Objects Categorisation procedure, which depends on hardware capabilities as well as their functionalities and location. For instance, consumer objects have very powerful resources and can be used in multiple purposes (e.g., control other objects), while smart detectors have very limited resources, and their main objectives are to detect changes inside the smart home (e.g., detect motion), also depicted in Figure 3.

### 4.2. Step 2

Next case-specific procedure to be followed is stemming from Phase 3 of the SLC Framework. Here each object is assigned an SLC. Considering the grouping schema proposed in previous step, objects belonging to the same group can have different SLC. For instance, smart detectors will have SLC1, whereas camera objects have SLC2 or SLC3. The process of assigning SLCs for each group depends on many factors: (i) hardware capabilities, and (ii) location at which such objects are being deployed.

To this end, smart detectors, for example, are equipped with SLC1, since such objects have very limited resources, no direct Internet access, are deployed inside homes, and, more importantly, have weak protection measures (MT1 and MT8). In contrast, consumer objects in smart homes according to our framework have either SLC4 for smart phones or SLC5 for laptops. This is because such objects (e.g., phones and laptops) have very powerful resources, and they are equipped with most of our suggested mitigation techniques. More importantly, objects with SLC4 have unique responsibilities compared to objects with SLC5. Objects with SLC5 are equipped with blockchain solutions to register and track SLCs for all smart home objects, detect fake SLCs, maintain a revocation list, and integrate the legacy objects into smart home, while objects with SLC4 are responsible for managing firmware updates of the all smart home objects, as they have required protection measures to do so.

It is worth mentioning that smart home objects in SLC Framework are not only used to achieve their specific tasks, but also to carry out other responsibilities due to their SLCs. For example, the main purpose for a smart TV is to allow its users, without the need to connect the TV antenna, to access to several channels which provide movies, music, and programs. Apart from providing such specific task, the smart TV has other responsibilities, such as managing communication between objects with SLC2 (e.g., smart heath objects) and the Internet, since the TV has required techniques to carry out such responsibilities.

Table 13 summarises the process of classifying smart home objects into different categories, along with their mitigation techniques required for each SLC. It is worth noting the assumption on the hardware capabilities of each smart home classification in this case study such as smart detectors, smart health, and customer objects. One can note that all smart home objects have a set of mitigation techniques through which previously mentioned attacks against IoT such as AT1, AT2, AT3, and AT5 are mitigated. A detailed explanation of how the framework will lesson such attacks is presented in Section 5.2.

### 4.3. Step 3

This step is dedicated to the definition of a secure communication plan among smart home objects, the main purpose of which is to prevent unexpected use of smart home data. Although smart home objects are classified into several groups, such objects are able to interact with each according to the suggested communication plan, which depends entirely on objects’ SLCs. For example, smart detectors equipped with SLC1 are able to communicate with indoor security cameras, energy and lighting, smart health, and household appliance, as long as such objects are located within the coverage area of smart detectors signals. Nevertheless, smart detectors can not communicate with smart phones and tablets, laptops, gateways, and the Internet.

An overview of the communication plan in which smart home objects can communicate securely with each other, the Internet, and legacy objects is presented in Table 14.

### 4.4. Step 4

Finally, the last step is dedicated to the illustration on the smart home seamless and secure integration of the legacy objects. A legacy object inside the smart home will attempt to interact with any objects such as gateways and consumer objects in smart home except objects with SLC1 and SLC 2 (e.g., smart detectors and smart health) due to their limited communication plan. However, the legacy objects are not able to communicate with smart home objects with SLC3 (home entertainment), SLC4 (phones and tablets), and SLC5 (laptops), unless they first communicate with objects with SLC5 and then receive their SLCs from them.

## 5. Discussion and Future Work

A summary of the previously mentioned research proposals is presented in Table 4, along with our intended objectives. It is not so difficult to recognise their limitations while going through them. This article, therefore, is directed to overcome those shortcomings that can be categorised as follows: (i) the lack of a thorough set of security and privacy guidelines for IoT assets, (ii) the absence of proper mitigation techniques to carry out such guidelines, (iii) the need of attack investigations related to IoT systems, (iv) the necessity of mitigation techniques classification as well as IoT objects classification, and (v) the need of a communication plan so that IoT objects will be able to communicate securely with each other or with the Internet.

In what follows, an illustration on how SLC Framework alleviates the attacks and threats against IoT and solves some IoT security challenges.

### 5.1. Analysis on IoT Security Challenges

In this part, qualitative arguments are offered to illustrate how the proposed framework can be used to address previously discussed IoT security challenges.

Lack of a secure development (SC1): SLC Framework’s Phase 2 is explicitly addressing this challenge. More specifically, a set of security and privacy guidelines is proposed, covering all IoT assets (physical objects, protocols, data at rest, and software), along with their appropriate mitigation implementation techniques. Integrating such guidelines and techniques by developers or manufacturers into their IoT systems or products from the early stages of development (life cycles) will lead to develop secure system, which in turn improves security and privacy by design for IoT.

Tight resource constraints (SC2): it is unrealistic to assign common protection measures for IoT objects, since such objects may come in different sizes, varying from resource-limited objects such as motion detectors to resource-rich ones such as smart phones. Smart phones, for example, can implement traditional security mechanisms, while it seems to be very difficult to apply such techniques in motion detectors without some modifications. To this point, the classification of objects into five categories, as per Phase 1 of the proposed framework is based on their hardware capabilities. Furthermore, different mitigation techniques are assigned accordingly in Phase 2 of the SLC Framework. For instance, objects in Category 1 implement only a few protection measures suitable to their limited resources, whereas objects in Category 5 implement almost all suggested protection measures due to their powerful hardware capabilities.

Designed for specific Tasks (SC3): being designed to carry out specific tasks and deployed in different environments, IoT objects require different mitigation techniques. In other words, it is wise to assign different protection measures to IoT objects based on their main functions or tasks. To this end, the proposed framework assigns different protection measures to IoT objects based on their tasks and hardware resources, as dictated by Phase 2 and Phase 3. For example, as the main goal of objects with SLC5 is to register and keep track of all SLCs, such objects thus are equipped with blockchain solutions to do so. In contrary, objects with SLC1, SLC2, and SLC3 are not armed with such solutions, as they are not designed to accomplish such goal.

Update mechanisms (SC5): as the security of IoT objects relies on a method in which such objects receive their newly released updates either locally or remotely, the proposed framework thus assigns different mitigation techniques for IoT objects as per Phase 3. For example, objects with SLC1 will not have firmware update methods, as they depend entirely on objects with SLC2 to update their firmware, while other objects with SLC2, SLC3, SLC4, and SLC5 will be equipped with such techniques to independently update their firmware.

Objects’ mobility (SC6): since the location of IoT objects either static or dynamic plays a key role in defining their security requirements, our framework therefore assigns different mitigation techniques for such objects based on their mobility. For instance, objects with SLC1 and SLC2 have a few protection measures, as they always interact with each other or with objects through SLC3. On the other hand, objects with SLC3, SLC4, and SLC5 have more mitigation techniques due to their communication with each other, the Internet, and legacy objects.

Uncontrolled environment (SC8): the environment at which IoT objects will be deployed and operated plays a key role in determining their proper mitigation techniques. To this point, IoT objects are classified broadly into two groups: objects operating in controlled environments and objects operating in uncontrolled areas. Thus, objects operating in controlled areas such as objects with SLC1 and SLC2 have a few mitigation techniques, as such objects always are deployed in attended areas and are monitored by human beings or security cameras to prevent physical attacks. In contrast, objects with SLC3, SLC4, and SLC5 have more protection measures to prevent physical attacks, as such objects may be deployed in unattended environments.

Although most of IoT security challenges presented in Section 2.1 have been addressed in our suggested framework, two security challenges, namely *(SC4)* ‘Changes in security requirements’ and *(SC7)* ‘The importance of IoT objects’, are left untouched. We do believe that such challenges can be addressed by developers or software engineers during the analysis phase of an IoT system’s requirements.

### 5.2. Attack and Threats Mitigation by SLC Framework

The proposed framework that addresses the most important IoT-specific attacks and threats and a brief analysis for each previously presented attack in Section 2.2 is discussed in the next paragraphs.

Eavesdropping (AT1): to mitigate such attacks, our framework prevents any object from sending and receiving its data or packets over unencrypted channels. This can be observed through mitigation techniques included in all suggested SLCs. For instance, objects with SLC1 implement DLLS to send/receive their data in encrypted format to/from objects with SLC2. Similarly, objects with SLC2, SLC3, SLC4, and SLC5 implement either Transport layer security or Network layer security to provide end-to-end secure communication channels between them.

Physical attacks (At2): to lessen this type of attacks, SLC Framework divides its objects based on their environments into two categories: controlled and uncontrolled environments. Objects with SLC1 and SLC2 always are deployed in controlled areas to prevent physical attacks despite not having mitigation techniques to do so, as such objects are always monitored by either people or security cameras. On the other hand, objects with SLC3, SLC4, and SLC5 can be deployed in uncontrolled environments, as they are equipped with hardware-based solutions such as tamper-proofing techniques to mitigate physical attacks.

Side-channel attacks (AT3): to alleviate such attacks, our framework integrates side-channel protection techniques into objects with SLC3, SLC4, and SLC5, whereas objects with SLC1 and SLC2 are be vulnerable to side-channel attacks. However, this is not an issue as these objects are not connected directly to the Internet, nor they are deployed in uncontrolled environments, according to the proposed methodology.

Malicious object insertion (AT4): to this end, the suggested framework forces its objects with different SLCs to first register their SLCs in objects with SLC5. It is worth repeating that objects with SLC5 are shielded with blockchain-based solutions so that other objects such as objects with SLC2, SLC3, and SLC4 are able to track all registered objects and therefore detect the malicious ones. For example, suppose that an object with fake SLC3 tries to communicate with an object with SLC2. The object with SLC2 have to check if the object is trying to communicate with has a genuine SLC3 by contacting any object with SLC5. If not, which is the case in this example, the object with SLC2 will not communicate with it and will notify any object with SLC5 about this incident.

Routing attacks (AT5): to lessen such attacks, the proposed framework compels the majority of its objects to apply Network layer security to prevent such attacks. For instance, objects with SLC2, SLC3, SLC4, and SLC5 have such mitigation techniques against routing attacks. In contrast, objects with SLC1 are vulnerable to such attacks, as they only implement two mitigation techniques. However, the suggested communication plan plays a key role to mitigate such threat, as it restricts the communication of objects with SLC1 to only objects with SLC2. More importantly, communication links or channels between objects with SLC1 and object with SLC 2 are encrypted using link layer security.

Malicious firmware (AT6): to mitigate this types of attacks, SLC Framework utilises blockchain-based solutions (e.g., pushed-based firmware update proposed in [86]) to update their objects securely. Manufacturers, by implemented the framework, will be able to build smart contracts for newly developed firmware versions and will push them to all objects with SLC4. During the update process, some objects with SLC4, called miners, will verify the integrity of pushed firmware, as they will be equipped with consensus protocol. Once the smart contracts are verified by miners, objects with SLC2, SLC3, and SLC5 will be able to send requests to objects with SLC4 and therefore download the latest versions of firmware available for them.

### 5.3. Limitations of the Study

The risks associated with the insider threats in our framework can be recognised in two processes: issuing and assigning SLCs and firmware updates. As the process of assigning and issuing SLCs depends heavily on entities (e.g, developers or manufacturers) implementing the framework, it is therefore vulnerable to malicious insider threats. It is possible that a developer who is responsible for issuing SLCs could accidentally or intentionally either alter the setting of SLCs or assign SLCs to wrong IoT objects. This is because security of our methodology relies heavily on its communication plan, which in turn depends on SLCs. The object with SLC1 is always connected to objects with SLC2, and is deployed in controlled areas, whereas the object with SLC3 communicates with all objects (except object with SLC1) and is deployed in uncontrolled environments.

Similarly, the blockchain-based firmware update scheme (smart contract and consensus mechanism) utilised in our framework is also susceptible to malicious insider threats despite its benefits in terms of verifying the firmware integrity and preventing DoS attacks. This is because our methodology assumes that all newly released firmware updates are pushed or published by a trustworthy manufacturer. However, this is not always the case because of two reasons. One is that the manufacturer may be compromised by an attacker, and therefore he could use manufacturer’s private keys to sign updates and push them into objects with SLC4. The other reason is that an employee at manufacturer, attacker, may be able (due to given access rights) to send malicious updates from the manufacturer’s server to all objects with SLC5.

### 5.4. Future Work

As the security requirements within an IoT network may change over time, the proposed framework should be able to adapt accordingly. While this is ongoing work, the current strategy is to use SLC5-enabled IoT devices as servers for certificate validation and device management functions. It ensures the highest level of security and eliminates the risk of a single point of failure. As security requirements change over time, these servers can be used to update and maintain the security of connected devices, ensuring that they remain secure and up to date. Overall, this approach would provide a scalable and efficient solution for managing the security of large-scale IoT deployments. The development of new communication techniques and attack methods is a constant concern for IoT security. To address this, dynamic SLC assignment and machine learning algorithms can be used to enhance the security posture of IoT systems. Dynamic SLC assignment assigns SLCs to IoT objects based on their current security state, ensuring that each object is given the appropriate SLC based on its capabilities and protection measures. Additionally, machine learning algorithms can be incorporated to continuously monitor the IoT system for new threats and adapt the security framework accordingly. This approach ensures that the framework remains up-to-date and effective in addressing new types of attacks.

## 6. Conclusions

The main goal of this paper is to develop a secure framework for IoT that allows different IoT objects to communicate securely with each other or with the Internet based on their SLCs. First, a classification of IoT objects into five categories based on their hardware capabilities is performed. Objects in Category 1 indicate that they have very limited resources, whereas objects in Category 5 have very powerful hardware capabilities. Second, the mitigation techniques are mapped for different types of objects with the layered approach. Third, each IoT object is assigned SLC, based on their hardware capabilities. SLC1 indicates that object has weak protection measures, while SLC5 implies strong protection measures implemented. Fourth, a communication plan that allows objects not only to communicate securely with each other but also with the Internet is proposed. Moreover, such a plan prevents unexpected use of IoT data. The integration approach of the SLC Framework with legacy objects is concluding the methodology. The proposed framework also can be used to address IoT-specific attacks and solve some of IoT security challenges. To demonstrate the feasibility and application of the suggested framework, an exemplification of the smart-home use case is provided. The future efforts will be focused on the protection against an insider attacker, since it is the main threat to the presented methodology. Moreover, penetration tests on the actual IoT objects equipped with the proposed SLCs are envisioned to evaluate the performance penalty as well as security benefits of using such framework.

## Figures and Tables

**Table 1:** IoT Security goals as defined by IAS octave.

Security Requirements	Definition	Abbreviations
Confidentiality	Only authorised objects or users can obtain access to the data	CONF
Integrity	Data completeness and accuracy is preserved	INTG
Non-repudiation	IoT system can validate the occurrence of any event	NREP
Availability	Ensuring accessibility of an IoT system and its services	AVAL
Privacy	Presence of privacy rules or policies	PRIV
Auditability	Monitoring of the IoT object activity	AUDI
Accountability	End users can take charge of their actions	ACNT
Trustworthiness	Reliability on IoT object identity	TRST

**Table 2:** Violated security goals per attack.

Attack ID	CONF	INTG	NREP	AVAL	PRIV	AUDI	ACNT	TRST
AT1	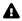		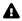		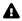			
AT2	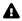	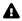	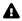	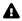	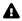	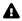	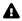	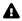
AT3	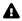	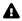			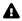			
AT4	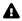	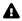	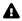	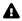	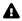	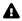	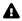	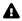
AT5	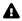	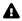	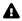		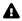		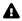	
AT6	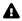	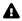	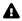	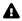	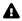	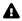	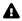	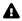

**Table 3:** Attack vector correlation to mitigation techniques.

ID	Mitigation Techniques	Attack IDs
MT1	Link layer security	AT1
MT8	Secure bootstrapping	AT1
MT2	Transport layer security	AT1, AT3, AT5
MT3	Network layer security	AT1, AT5
MT4	Firmware update methods	AT6
MT15	Searchable encryption	AT1,
MT10	Hardware-based solutions	AT2, AT4, AT6
MT6	Side channel protection	AT3
MT18	Access control methods	AT2, AT3, AT4
MT19	Secure IoT OSs	AT6
MT20	SDN-based solutions	AT1, AT4, AT5
MT21	Application layer security	AT1, AT3, AT6
MT22	Cryptographic schemes	AT2, AT3, AT4
MT9	Blockchain solutions	AT1, AT5, AT6
MT14	Secure storage schemes	AT1, AT2
MT13	Transient data storage	AT5
MT7	Decommissioning methods	AT2
MT11	Deduplication schemes	AT1, AT2, AT4
MT12	Anonymisation schemes	AT4, AT6
MT16	Monitoring and auditing	AT2, AT4, AT6
MT17	Recovery strategy	AT6
MT5	Intrusion detection system	AT1, AT4, AT5, AT6

**Table 4:** Comparison of research efforts presented in the literature.

Addressed Features		State-of-the Art Work									
		[63]	[64]	[65]	[32]	[66]	[19]	[23]	[14]	[15]	This Work
Addressed IoT SCs	SC1	✓	✓	✓	✓	✓	✓	✓	✓	✓	✓
	SC2	✗	✗	✗	✗	✓	✓	✓	✓	✓	✓
	SC3	✓	✗	✗	✗	✗	✗	✗	✓	✓	✓
	SC4	✗	✗	✗	✗	✗	✗	✗	✓	✓	✗
	SC5	✓	✓	✓	✗	✓	✓	✓	✓	✓	✓
	SC6	✗	✗	✗	✗	✗	✓	✗	✓	✓	✓
	SC7	✗	✗	✗	✗	✗	✗	✗	✓	✓	✗
	SC8	✗	✗	✗	✗	✗	✓	✗	✓	✓	✓
Addressed IoT attacks	AT1	-	-	-	-	-	✓	✓	-	-	✓
	AT2	-	-	-	-	-	✓	✓	-	-	✓
	AT3	-	-	-	-	-	✓	✗	-	-	✓
	AT4	-	-	-	-	-	✓	✗	-	-	✓
	AT5	-	-	-	-	-	✓	✗	-	-	✓
	AT6	-	-	-	-	-	✓	✓	-	-	✓
Types of guidelines	Privacy	✓	✓	✓	✗	✓	✓	✗	✗	✗	✓
	Security	✗	✓	✓	✓	✓	✓	✓	✗	✗	✓
Communication Plan		✗	✗	✗	✗	✗	✗	✗	✗	✗	✓
Objects classification		✗	✗	✗	✗	✗	✗	✓	✓	✓	✓
Protection measures classification		✗	✗	✗	✗	✗	✗	✓	✗	✓	✓

The symbols ✓, ✗, and - indicate the addressed, not addressed, and not mentioned features, respectively.

**Table 5:** Classification of IoT objects based on their hardware capabilities.

Object Categories	CPU/ Microcontroller	Memory (M)	On-Board Storage	Power Consumption (P)	Example
Category 1	Low CPU such as 8- bit Microcontroller 16 MHz	M ≤ 32 KB	None	P ≤ 1 W	Arduino Mego
Category 2	Moderate CPU such as 32- bit Microcontroller 80 MHz	32 KB < M ≤ 80 KB	None	P ≤ 1 W	NodeMCU ESP-12
Category 3	Single core CPU (e.g., ARM1176 single-core 1 GHz)	80 KB < M ≤ 512 MB	≤4 GB	1 W < P ≤ 2W	Raspberry Pi Zero
Category 4	Quad core CPU (e.g., ARM Cortex-A53 quad-core 1.2 GH)	512 MB < M ≤ 2 GB	≤8 GB	2 W < P ≤ 4 W	Raspberry Pi 3
Category 5	High (e.g., ARM Cortex-A57 quad-core 2 GHz)	M ≥ 8 GB	High (≥32 GB)	High	NVIDIA Jetson TX2

**Table 6:** A summary of the suggested guidelines for physical layer along with their countermeasures.

Guidelines	Reasoning	Implementation Techniques
Secure boot process	Fixed hardware secure boot process prevents running a malicious code	MT22: Cryptographic schemes [70], MT10: Hardware-based solutions [49,71]
Update firmware securely	Secure firmware update enables detection of a malicious firmware	MT4: Firmware update methods [72]
Use hardware identifier	Unique identifier can serve as a proof of origin and permit threat source attribution	MT10: Hardware-based solutions
Prevent physical tampering	A tamper-resistant measure prevents physical attacks	MT10: Hardware-based solutions (e.g., tamper-proofing techniques)
Safe disposal	Destruct data properly when reaching end-of-life stage	MT7: Decommissioning methods [43]
Implement hardware trust	A hardware trust method prevents malicious modification of data	MT10: Hardware-based solutions (e.g., a PUF-based authentication [73])
Detect abnormal nodes	Attackers may modify behaviour of the IoT object for various purposes	MT5: IDS techniques (e.g., a method based on Markov model [41])
Prevent node replication	Safeguard object’s identification number	MT22: Cryptographic schemes
Prevent unwanted IC modifications	Detect malicious modifications on its IC	MT6: Side channel analysis (e.g., dynamic permutation [74]), MT10: Hardware-based solutions

**Table 7:** A summary of the suggested guidelines for communication layer along with their countermeasures.

Guidelines	Reasoning	Implementation Techniques
Implement hop to hop security	Encrypt and decrypt packets to the next object through shared keys	MT1: DLLS such as IEEE 802.15.4 [75]
Secure bootstrapping	Exchange of network settings, such as link-layer encryption keys, network names, and wireless channels	MT8: Diet HIP [45]
Prevent packet modification	Message integrity mechanism to prevent packet injection attacks	MT2: Transport layer security [8], MT3: Network layer security [76], MT21: Application layer security [77]
Encrypt data communication	Prevent unauthorised access to sensitive data	MT2: Transport layer security, MT3: Network layer security, MT9: Blockchain solutions [78], MT20: SDN-based solutions in [79]
Support end to end security	Prevent unauthorised access to sensitive data	MT2: Transport layer security, MT3: Network layer security, MT9: Blockchain solutions
Prevent packet duplication	Prevent reply attacks	MT2: Transport layer security, MT3: Network layer security, MT21: Application layer security
Strong key management	Protect data during communication	MT20: SDN-based solutions, MT9: Blockchain solutions, MT2: Transport layer security, MT21: Application layer security
Hidden data routing	Anonymous routing methods to protect routing data	MT20: SDN-based solutions, M9: Blockchain solutions, MT3: Network layer security
Ensure continuous monitoring	Monitor abnormal activities	MT5: IDS techniques

**Table 8:** A summary of the suggested guidelines for data at rest layer along with their countermeasures.

Guidelines	Reasoning	Implementation Techniques
Minimise data storage	Reduce IoT breaches by deleting any portion of data not required to achieve a certain task	MT11: Deduplication [80], MT12: Anonymisation schemes [81]
Minimise data retention	Prevent data breaches	MT13: Transient data storage [54]
Encrypt data storage	Avoid data leakage	MT14: Secure storage schemes [82], MT15: Searchable encryption [83]
Prevent data leakage	Data are vulnerable to side channel attacks	MT16: Monitoring and auditing, MT15: Searchable encryption, MT12: Anonymisation schemes, MT13: Transient data storage
Ensure data availability	Ensure functioning of the data dependent applications	MT11: Deduplication schemes, MT17: Recovery strategy [59]
Ensure authorised access	Prevent unauthorised access	MT18: Physical security [84]
Remove or hide sensitive data	Identity protection	MT12: Anonymisation schemes
Search on encrypted data	Application functionality over encrypted data	MT15: Searchable encryption
Proper data destruction	Prevent data leakage	MT7: Decommissioning

**Table 9:** A summary of the suggested guidelines for software layer along with their countermeasures.

Guidelines	Reasoning	Implementation Techniques
Prevent malicious requests	Defence in depth	MT18: Access control methods
Integrate OS with network security	To offer data integrity and privacy	MT19: Secure IoT OSs
Provide memory protection	Strong process management to manage resources	MT19: Secure IoT OSs
Validate and encrypt updates	Prevent the injection of malware during the update	MT19: Secure IoT OSs, MT4: Secure update methods
Provide events trace	Continuously monitor logs, processes and software	MT16: Monitoring and auditing
Provide memory protection	Properly allocate/deallocate memory for different threads and processes	MT19: Secure IoT OSs

**Table 10:** SLCs classification according to the implementation of the mitigation techniques.

ID	Mitigation Techniques	SLC1	SLC2	SLC3	SLC4	SLC5
MT1	Link layer security	✓	✓	✓	✓	✓
MT8	Secure bootstrapping	✓	✓	✓	✓	✓
MT2	Transport layer security	✗	✓	✓	✓	✓
MT3	Network layer security	✗	✓	✓	✓	✓
MT4	Firmware update methods	✗	✓	✓	✓	✓
MT15	Searchable encryption	✗	✗	✓	✓	✓
MT10	Hardware-based solutions	✗	✗	✓	✓	✓
MT6	Side channel protection	✗	✗	✓	✓	✓
MT18	Access control methods	✗	✗	✓	✓	✓
MT19	Secure IoT OSs	✗	✗	✓	✓	✓
MT20	SDN-based solutions	✗	✗	✓	✓	✓
MT21	Application layer security	✗	✗	✓	✓	✓
MT22	Cryptographic schemes	✗	✗	✓	✓	✓
MT9	Blockchain solutions	✗	✗	✗	✓	✓
MT14	Secure storage schemes	✗	✗	✗	✓	✓
MT13	Transient data storage	✗	✗	✓	✓	✗
MT7	Decommissioning methods	✗	✗	✗	✗	✓
MT11	Deduplication schemes	✗	✗	✗	✗	✓
MT12	Anonymisation schemes	✗	✗	✗	✗	✓
MT16	Monitoring and auditing	✗	✗	✗	✗	✓
MT17	Recovery strategy	✗	✗	✗	✗	✓
MT5	Intrusion detection system	✗	✗	✓	✗	✗

The symbols ✓ and ✗ indicate the included and not included MTs, respectively.

**Table 11:** Assigning SLCs to IoT objects.

	SLC1	SLC2	SLC3	SLC4	SLC5
Category 1	✓	✓	✗	✗	✗
Category 2	✓	✓	✗	✗	✗
Category 3	✓	✓	✓	✗	✗
Category 4	✓	✓	✓	✓	✗
Category 5	✓	✓	✓	✓	✓

The symbols ✓ and ✗ indicate suitable and not suitable SLC level, respectively.

**Table 12:** The suggested communication plan.

	SLC1	SLC2	SLC3	SLC4	SLC5	Internet
**SLC1**	✗	✓	✗	✗	✗	✗
**SLC2**	✓	✓	✓	✓	✓	✗
**SLC3**	✗	✓	✓	✓	✓	✓
**SLC4**	✗	✓	✓	✓	✓	✓
**SLC5**	✗	✓	✓	✓	✓	✓

The symbols ✓ and ✗ indicate possible and not possible communication between SLC levels, respectively.

**Table 13:** A summary of smart home object classification, along with SLCs.

Object Category	Example	Object Class	SLC Type	Mitigation Techniques
Smart detectors	Smoke, gas, motion, alarm	Category 1	SLC1	MT1 and MT8
Security Cameras	Indoor	Category 2	SLC2	MT1, MT2, MT3, MT4, MT8
Security Cameras	Outdoor	Category 3	SLC3	MT1, MT2, MT3, MT4, MT5, MT6, MT8, MT10, MT13, MT15, MT18, MT19, MT20, MT21, MT22
Energy and lighting	Smart light, -switch, -plug, -outlet	Category 2	SLC2	MT1, MT2, MT3, MT4, MT8
Home entertainment	Home assistant, TV, music player	Category 3	SLC3	MT1, MT2, MT3, MT4, MT5, MT6, MT8, MT10, MT13, MT15, MT18, MT19, MT20, MT21, MT22
Home entertainment	Smart remote	Category 2	SLC2	MT1, MT2, MT3, MT4, MT8
Smart health	Blood pressure, wrist band, smart watch, body media	Category 2	SLC2	MT1, MT2, MT3, MT4, MT8
Household appliances	Fridge, coffee maker, blender, microwave	Category 2	SLC2	MT1, MT2, MT3, MT4, MT8
Consumer objects	Smart phone, tablet	Category 4	SLC4	MT1, MT2, MT3, MT4, MT6, MT8, MT9, MT10, MT13, MT14, MT15, MT18, MT19, MT20, MT21, MT22
Consumer objects	Laptops, computers	Category 5	SLC5	MT1, MT2, MT3, MT4, MT6, MT7, MT8, MT9, MT10, MT11, MT12, MT14, MT15, MT16, MT17, MT18, MT19, MT20, MT21, MT22
Gateways	Router, hub	Category 3	SLC3	MT1, MT2, MT3, MT4, MT5, MT6, MT8, MT10, MT13, MT15, MT18, MT19, MT20, MT21, MT22

**Table 14:** The suggested communication plan for smart home objects.

	Smart Detectors	Indoor Security Cameras	Outdoor Security Cameras	Energy and Lighting	Smart Health	Household Appliance	Smart Phones/ Tablets	Laptops/ Computers	Gateways	Internet	Legacy Objects
**Smart detectors**	✗	✓	✗	✓	✓	✓	✗	✗	✗	✗	✗
**Indoor security cameras**	✓	✓	✓	✓	✓	✓	✓	✓	✓	✗	✗
**Outdoor security cameras**	✗	✓	✓	✓	✓	✓	✓	✓	✓	✓	✓
**Energy and lighting**	✓	✓	✓	✓	✓	✓	✓	✓	✓	✗	✗
**Smart health**	✓	✓	✓	✓	✓	✓	✓	✓	✓	✗	✗
**Household appliance**	✓	✓	✓	✓	✓	✓	✓	✓	✓	✗	✗
**Smart phones/tablets**	✗	✓	✓	✓	✓	✓	✓	✓	✓	✓	✓
**Laptops/computers**	✗	✓	✓	✓	✓	✓	✓	✓	✓	✓	✓
**Gateways**	✗	✓	✓	✓	✓	✓	✓	✓	✓	✓	✓

The symbols ✓ and ✗ indicate possible and not possible communication, respectively.

## Data Availability

Not applicable.

## Abbreviations

The following abbreviations are used in this manuscript:

6LoWPAN	IPv6 networking for Low power Wireless Personal Area Networks
ABE	Attribute-Based Encryption
AES	Advanced Encryption Standard
AMQP	Advanced Message Queuing Protocol
BITAG	Broadband Internet Technical Advisory Group
CIA	Confidentiality, Integrity, and Availability
CoAP	Constrained Application Protocol
CPU	Central Processing Unit
CSS	Cloud Storage Service
DAC	Discretionary Access Control
DDS	Data Distribution Service
DLLS	Data Link Layer Security
DoS	Denial of Service
DTLS	Datagram Transport Layer security
HIP	Host Identity Protocol
IAS	Information, Assurance, and Security
IC	Integrated Circuit
IDS	Intrusion Detection System
IoT	Internet of Things
IoTSF	IoT Security Foundation
LLN	Low-Power and Lossy Network
MAC	Mandatory Access Control
MQTT	MQ Telemetry Transport
OS	Operating System
OTA	Over-the-air
OWASP	Open Web Application Security Project
PUF	Physical Unclonable Function
RBAC	Role-Based Access Control
RFID	Radio Frequency Identification
RM	Reference Model
RPL	Routing Protocol for Low-Power and Lossy Networks
SDN	Software Defined Network
SE	Searchable Encryption
SLC	Security Level Certificate
SLC	Framework Security Level Certificates Framework
SQL	Structured Query Language
SSL	Secure Sockets Layer
TLS	Transport Layer Security
WSN	Wireless Sensor Network
XMPP	Extensible Messaging and Presence Protocol
